# Rice-*Magnaporthe* transcriptomics reveals host defense activation induced by red seaweed-biostimulant in rice plants

**DOI:** 10.3389/fgene.2023.1132561

**Published:** 2023-06-23

**Authors:** Sahana N. Banakar, M. K. Prasannakumar, P. Buela Parivallal, D. Pramesh, H. B. Mahesh, Aditya N. Sarangi, M. E. Puneeth, Swathi S. Patil

**Affiliations:** ^1^ Plant PathoGenOmics Laboratory, Department of Plant Pathology, University of Agricultural Sciences, Bengaluru, India; ^2^ Rice Pathology Laboratory, All India Coordinated Rice Improvement Programme, University of Agricultural Sciences, Raichur, India; ^3^ Department of Genetics and Plant Breeding, College of Agriculture, Mandya, India; ^4^ BaseSolve Informatics Pvt. Ltd., Ahmedabad, Gujarat, India

**Keywords:** rice, blast disease, *Magnaporthe oryzae*, seaweed, biostimulants, transcriptomics

## Abstract

Red seaweed extracts have been shown to trigger the biotic stress tolerance in several crops. However, reports on transcriptional modifications in plants treated with seaweed biostimulant are limited. To understand the specific response of rice to blast disease in seaweed-biostimulant-primed and non-primed plants, transcriptomics of a susceptible rice cultivar IR-64 was carried out at zero and 48 h post inoculation with *Magnaporthe oryzae* (strain MG-01). A total of 3498 differentially expressed genes (DEGs) were identified; 1116 DEGs were explicitly regulated in pathogen-inoculated treatments. Functional analysis showed that most DEGs were involved in metabolism, transport, signaling, and defense. In a glass house, artificial inoculation of MG-01 on seaweed-primed plants resulted in the restricted spread of the pathogen leading to the confined blast disease lesions, primarily attributed to reactive oxygen species (ROS) accumulation. The DEGs in the primed plants were defense-related transcription factors, kinases, pathogenesis-related genes, peroxidases, and growth-related genes. The beta-D-xylosidase, a putative gene that helps in secondary cell wall reinforcement, was downregulated in non-primed plants, whereas it upregulated in the primed plants indicating its role in the host defense. Additionally, Phenylalanine ammonia-lyase, pathogenesis-related Bet-v-I family protein, chalcone synthase, chitinases, WRKY, AP2/ERF, and MYB families were upregulated in seaweed and challenge inoculated rice plants. Thus, our study shows that priming rice plants with seaweed bio-stimulants resulted in the induction of the defense in rice against blast disease. This phenomenon is contributed to early protection through ROS, protein kinase, accumulation of secondary metabolites, and cell wall strengthening.

## 1 Introduction

Rice is the most important crop, providing food to more than half of the world’s population. Different biotic and abiotic stresses worldwide cause up to 50% loss in crop yield. Among all, rice blast disease caused by *Magnaporthe oryzae* (*M. oryzae*) is one of the most serious diseases threatening rice productivity in almost all rice-growing regions of the world. It is responsible for several epidemics in tropical and subtropical countries (Li et al., 2021). Blast disease alone is responsible for the annual yield loss of 10%–30%, which can reach up to 100% under favorable conditions (Talbot, 2003; Asibi et al., 2019). Necrotic lesions due to blast disease on the rice leaf surface result in the reduction of photosynthetic rate, plant height, number of panicles, and grain weight. Another severe form of the disease, i.e., neck blast, leads to barren panicles and sterile grains, leading to complete yield loss, and thus it is a major threat to global rice production (Kumar et al., 2021). Employing novel ways to induce plant-defense-response systems to counteract the adverse effect of pathogens could be transformative for agriculture. With the onset of climate change, pesticide resistance, and the ever-growing human population, the need for new innovative agricultural practices is imminent. Plant bio-stimulants, such as those derived from seaweed extracts, have been unique and gaining more importance recently (Islam et al., 2020). Seaweeds are macroalgae that constitute an integral component of marine and coastal ecosystems, contributing to their rich biodiversity and the overall biosphere. Interestingly, seaweed biostimulants have reportedly been shown to contribute to plant growth promotion, increased yields, and plant tolerance to abiotic and biotic stresses. Seaweed extracts also elicit plant defense responses against plant pathogenic bacterial, fungal, and even viral pathogens, thereby protecting crops from significant economic damage from diseases (Ali and Ramsubhag, 2021). Over the decades, seaweed extracts have been highly explored for possible use in crop production to improve biomass yield and produce quality (Ali et al., 2019). Several reports suggest the control of fungal and bacterial diseases by applying seaweed extracts. The reduction of infection levels in seaweed extract-treated plants is due to induced systemic or systemic acquired resistance. Seaweed biostimulants enlisted a number of defense mechanisms in plants against biotic stresses (Pushp et al., 2019). Our previous study has reported the several polysaccharides in the cell wall of seaweeds, such as ulvans, laminarins, and carrageenans, and their derived oligosaccharides to confer some of these resistant responses in plants (Banakar et al., 2022). These bioactive molecules induce an oxidative burst and various defense pathways mediated through salicylic acid, jasmonic acid, and ethylene (Vera et al., 2011). This reaction cascade leads to the upregulation of pathogenesis-related proteins (PR proteins), various defense enzymes, and increased phenolic compounds (Sahana et al., 2022). Several methods have been employed to study rice and *M. oryzae* interaction (Mahesh et al., 2021). However, molecular mechanisms underlying at transcriptome level for seaweed-induced host defense are not yet completely understood. A genomic technique like RNA sequencing (RNA-Seq) has proved to be helpful in analyzing differentially expressed patterns of defense-related genes relieving the combined effect of transcription factors myeloblastosis family of transcription factors (MYB), WRKY transcription factors, ethylene response factor (ERF), signaling kinases, Reactive Oxygen Species (ROS) and cell wall modification-related genes, etc. Hence the current study aimed to determine the biological effect of seaweed biostimulant (LBD1) derived from red seaweed (*Kappaphycus* sp. and *Eucheuma* sp.) at the transcriptome level in rice- *Magnaporthe* pathosystem. In this study, we report the transcriptional regulation of defense against blast disease of rice in the red-seaweed derived biostimulant treated plants of rice cultivar IR-64.

## 2 Methods

### 2.1 Source of biostimulants

The seaweed biostimulant (LBD-1, 20% solid extract) used in the study was provided by Sea6Energy Pvt Ltd, Bengaluru, India. Briefly, the tropically grown red seaweed biomass of *Kappaphycus* sp and *Eucheuma* sp were processed by following two patented technologies (US10358391B2 and PCT/IN 2019/050831) to obtain solid and liquid fractions. The LBD-1 was prepared from solid fraction extracts rich in bioactive sulphated galacto oligosaccharides (Nori et al., 2017; Girish et al., 2020). The total Sodium (% w/w) content of LBD1 is 0.51 ± 0.05, estimated by the Inductively Coupled Plasma Optical Emission Spectroscopy (ICP-OES) technique.

### 2.2 Fungus, plant material, growth conditions, and treatments

Genetically pure seeds of rice cultivar IR-64, a commonly grown rice cultivar in India, that is, susceptible to blast disease, were collected from the All India Co-ordinated Rice Improvement Programme, ZARS, Mandya, India. The seeds were surface sterilized, germinated, and allowed to grow in a glass house facility (28°C ± 2°C and 16 h light/8 h dark) at the Department of Plant Pathology, University of Agricultural Sciences, Bangalore, India. These plants were then used for the inoculation of *M*. *oryzae* strain MG-01 obtained from the National Centre for Biological Sciences, Bengaluru. For inoculation, fungus culture was maintained on oatmeal agar for 2 weeks in the dark at 28 °C and then exposed to fluorescent lights for 1 week at room temperature for sporulation. With the aid of a hemocytometer, the spore concentration was set to 5 × 10^5^ spores ml^−1^. The punch inoculation method described by Ono et al., 2001 was used for artificial inoculation. The experiment was laid out with five treatments subjected to priming of three-week-old plants with biostimulants *viz*., 0 h (before priming or inoculation); MG-01 challenge inoculation without priming; T_1_, priming of plants with LBD1 (2 ml L^−1^) and challenge inoculation with MG-01; T_2_, LBD1 (2 ml L^−1^); T_3_, and water control; T_4_. The dose of the seaweed biostimulant was determined, as described in our previous study (Sahana et al., 2022). The inoculated plants were placed in the incubator at 25°C for 24 h and then moved to the mist chamber with the relative humidity maintained at 90%. After the inoculation, leaves were monitored every day for the development of necrotic lesions up to 6 days post inoculation (dpi). All the treatments were replicated thrice, and significant variations between the different treatments were calculated by two-way ANOVA (*p* < 0.05).

### 2.3 Effect of seaweed biostimulants on the production of ROS

The production of ROS was validated by *In vivo* quantification at 0 (before inoculation), 24, 48, and 72 h post inoculation (hpi) in the treatements mentioned above (T_1_-T_4_). Determination of the ROS *viz*., hydrogen peroxide (H_2_O_2_) and superoxide anion (O_2_^−^) radicals were performed by diaminobenzidine (DAB) Staining and histochemical detection techniques, respectively (Walker and Sunkar, 2010). All the treatments were replicated thrice, and significant variations between the different treatments were calculated by two-way ANOVA (*p* < 0.05).

### 2.4 RNA isolation and cDNA preparation

The time points for sample collection were determined based on symptom expression, where the necrotic lesions were visible on the leaves at 48 hpi. Thus, 48 hpi was considered an early infection response to study time-course transcriptome analysis and was compared with 0 h. All the samples were frozen in liquid nitrogen and stored at −80°C. Collected leaf samples of different treatments (0 h (before priming or inoculation); MG-01 challenge inoculation without priming; T_1_, priming of plants with LBD1 (2 mL L^−1^) and challenge inoculation with MG-01; T_2_, LBD1 (2 mL L^−1^); T_3_, and water control; T_4_) were used for total RNA isolation using an RNeasy plant mini kit (Qiagen, Germany) according to the manufacturer’s protocol. The isolated RNA was analyzed for its quality by gel electrophoresis and quantified by spectrophotometer (Nano-drop, Denovix, United States). For transcriptome analysis, samples were duplicated and outsourced to a commercial facility (AgriGenome Labs Pvt Ltd, Kochi, India). About 1 μg of total RNA, isolated from the samples, was used to prepare cDNA using a reverse transcription Kit (Qiagen, Germany) following the manufacturer’s instructions.

### 2.5 Library preparation, illumina sequencing, processing of the reads and analysis

Transcriptome sequencing and analysis were done using the Illumina Hiseq Platform. The comparison was made between T_1_ *vs*. T_2_, 0 h *vs*. T_1_, 0 h *vs*. T_2_, 0 h *vs*. T_3,_ and 0 h *vs*. T_4_. The parameters from fastq files were analyzed for reading quality check *viz.,* base quality score distribution, sequence quality score distribution, average base content per read, GC distribution in the reads, PCR amplification issue, and over-represented sequences. In the pre-processing step of the raw reads, the adaptor sequences and low-quality bases were trimmed using AdapterRemoval-v2 (version 2.2.0; Schubert and Orlando, 2016). From the preprocessed reads, ribosomal RNA sequences were removed by aligning the reads with the silva database using bowtie2 (version 2.2.9; Langmead and Salzberg, 2012) and subsequent workflow using samtools (version 0.1.19; Li et al., 2009), sambamba (version 0.6.7; Tarasov et al., 2015), BamUtil (version 1.0.13) tools and in-house scripts. The pre-processed and rRNA removed reads were aligned to the Rice Genome downloaded from MSU-7 (ftp://ftp.plantbiology.msu.edu/pub/data/Eukaryotic-_ Projects/o_sativa/annotation_dbs/pseudomolecules/version_7.0/all.dir). The alignment was performed using the STAR program (version 2.5.3a; Dobin et al., 2013). After aligning the reads, differential expression analysis was performed using cuffdiff program of cuffinks package (version 2.2.1; Trapnell et al., 2010). In cufflink, RNA-seq reads were accepted and assembled into a parsimonious set called transcripts. Lastly, Cuffdiff version 2.1.1 was used to quantify transcripts in terms of fragments per kilobase of transcript per million mapped reads (FPKM) and to obtain a list of the significant differentially expressed loci. Differentially expressed genes with fold change values of ≥ +2 and −2 and *p*-value of ≤ 0.05 were used to select the significant genes from the data. Gene ontology (GO) analysis was used to classify significant differentially expressed genes based on the molecular function, biological process, and cellular component. Plant Gene Sequence Enrichment Analysis **(**PlantGSEA) was carried out based on GO gene sets, Gene Family Based gene sets, Curated gene sets (PlantCyc gene sets, KEGG gene sets, Plant Ontology gene sets, References colleted gene sets), and Motif gene sets (MicroRNA Targets). This analysis was performed with significantly up and downregulated genes of the individual treatment combinations (Yi et al., 2013).

### 2.6 Validation of transcripts by quantitative real-time PCR (qRT-PCR)

Quantitative real-time PCR (CFX96, Bio-Rad) was performed using SYBR Green (TaKaRa) to validate the differentially expressed genes obtained from the RNA-seq data. According to the gene sequences in the gene database, primers were designed for 20 selected genes (Table 1). Three biological replicates were used for the experiment. The *18s* and ubiquitin were used as housekeeping genes for normalization. The relative expression was calculated using the comparative cycle threshold method (Livak and Schmittgen, 2001).

**TABLE 1:** qPCR validation of the selected genes which are up and downregulated in primed and non-primed plants.

SI. NO	Gene ID	Gene name	Forward primer (5′-3′)	Reverse primer (5′-3′)
1	LOC_Os01g72530	OsCML31 - Calmodulin-related calcium sensor protein	GGC​ATG​TAC​GAG​ATG​GAA​G	TTGAACTCGTCGAAGCTG
2	LOC_Os02g08440	WRKY71	TGGATCCGTGGATTAGCA	CAA​CCT​CTG​GGT​CTT​TCT​TG
3	LOC_Os02g47470	cytochrome P450	GAT​TGC​CAA​GGA​GAA​AGA​GG	GGA​TCA​GGT​ACC​CTT​GGT​AT
4	LOC_Os04g48290	MATE efflux family protein	CGTCATGGCATTCTGGTT	TGTCGTCACTCTCCATGT
5	LOC_Os01g01660	isoflavone reductase	AAGGTGGTGTTCGTGGAG	CACCCTCTCCAGCTTCTT
6	LOC_Os03g15370	ubiquitin fusion protein	AAC​ATC​CAG​AAG​GAG​TCC​A	TCTTGCGGCAGTTTGTAG
7	LOC_Os08g09060	Cupin domain containing protein	CTC​AAC​AAG​GGT​GAT​GTG​TT	TCT​GAA​ATC​GGT​GGC​TTT​G
8	LOC_Os04g56430	cysteine-rich receptor-like protein kinase	AGG​AGA​TCC​TCT​TCT​CCA​C	CGT​AGC​ACT​CGT​TGT​AGA​C
9	LOC_Os06g43304	cytochrome P450	AGAAGACTGCTCCATCGT	ATCAGTTCTGCCATTGCC
10	LOC_Os05g04500	peroxidase precursor	GTG​ATG​GCT​TCG​TCT​CTA​AG	GTACAGCCGGTTTGTGAA
11	LOC_Os08g36760	remorin C-terminal domain containing protein	TTG​ACC​ACC​AGC​AGT​AAA​C	CCA​TCT​GCA​CAT​CCC​TTA​TC
12	LOC_Os02g08440	WRKY71	TGGATCCGTGGATTAGCA	CAA​CCT​CTG​GGT​CTT​TCT​TG
13	LOC_Os02g04130	DUF1645 domain containing protein	CGC​CAT​CAT​CCA​AAC​TGA​A	TGGCCTTGCTGTAGAAGA
14	LOC_Os03g08330	ZIM domain containing protein	GCT​GAC​CAT​CTT​CTA​CGG​T	TCCTCATGATGGGCATGT
15	LOC_Os07g12340	NAC domain-containing protein 67	TCA​AGA​CGG​AGT​GGA​TCA​T	TCCTGCTCTGCATCTTCT
16	LOC_Os06g44010	WRKY28	CGA​TTA​AGG​TTC​TCG​TGG​AG	CAT​CAT​GTC​GTT​GAC​CTG​T
17	LOC_Os04g52090	AP2 domain containing protein	AGC​CGT​TCC​TGT​TCC​TCG​AC	CGT​CCA​CCA​CGG​ATG​ACG​A
18	LOC_Os08g36480	nitrate reductase	CATCATGCTCGCCTACAT	CGCGGTTGTCCTTGTAAT
19	LOC_Os05g24650	DUF567 domain containing protein	CTG​TTC​AAC​GGG​AAG​GGG​TT	TAG​ATG​AGC​CAC​TCG​TCC​GC
20	LOC_Os11g29720	cytochrome P450	GAC​TAC​GGC​AAT​GGT​GAA​TA	TAG​ATA​GGC​AGT​GGA​GAG​AC
21	OsUBQ	Ubiquitine	GAC​CAG​CAG​CGG​CTG​ATC​TTC	CTC​AGC​TGG​TTG​CTG​TGA​CCA​C
22	18s	18s ribosomal RNA	CGC​GCG​CTA​CAC​TGA​TGT​ATT​CAA	TAC​AAA​GGG​CAG​GGA​CGT​AGT​CAA

### 2.7 Microplot experiment

To study the effect of seaweed biostimulant in natural conditions, a micro plot (5 sq mt) experiment was laid out with three different treatments, i.e., Tricyclazole (Recommended fungicide against blast disease); T_1_, LBD1; T_2_ and water control; T_3_ with artificial inoculation of the conidial suspension at the tillering stage of the crop. The experiment was conducted using rice cultivar IR-64 at ZARS, Mandya, India. From each trial plot of a 5-m square, an area of 1 m^2^ was randomly selected for disease observation. A total of three sprays were taken at 15 days intervals. After the first spray (30 days after transplanting), plots were observed periodically until harvest to determine the severity of the leaf blast disease. The neck blast disease incidence was recorded at the time of harvest. Ten hills were selected randomly to assess the severity of the disease. The disease was assessed using the 0–9 scale (Standard Evaluation System, 1996), and resultant disease grades were converted into percent disease index (PDI) using a formula given by Wheeler (1969). Individual plots were harvested, and the grain and straw yields were determined using an electronic balance*.* All the treatments were replicated thrice, and significant variations between the different treatments were calculated by one and two-way ANOVA (*p* < 0.05).

## 3 Results

### 3.1 Effect of seaweed bioformulation on blast disease

**FIGURE 1:**
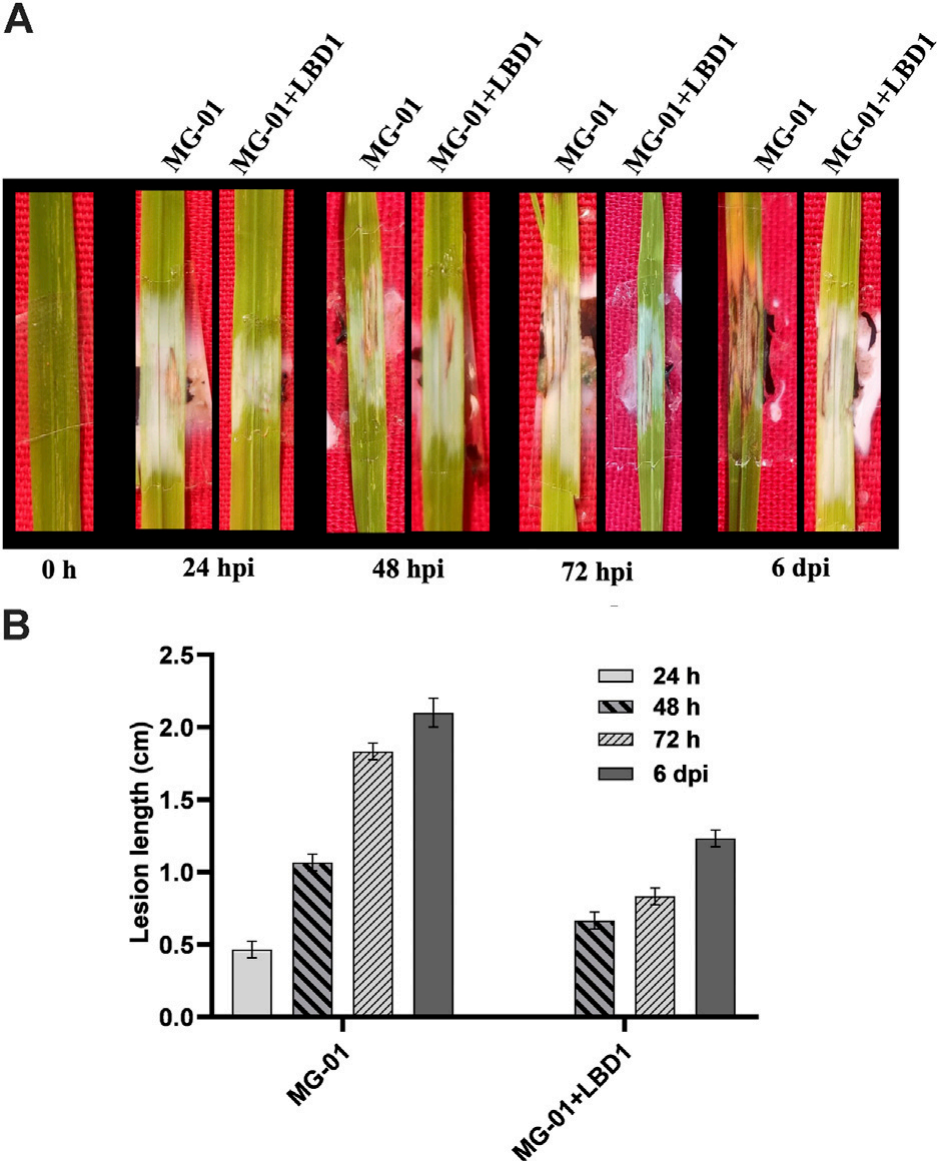
Challenge inoculation of MG-01 in primed and non-primed plants. Images of the rice leaves showing the impact of seaweed priming at different time intervals **(A)** Graphical representation of the lesion length in primed and non-primed plants at different intervals **(B)** where at 24 h, the LBD1 treated plants did not show any symptoms.

The rice cultivar IR-64, inoculated with MG-01, was observed for the disease symptoms at different time points in primed and non-primed plants. Plants in water control showed only wounding marks even after 6 dpi. At 24 hpi, primed plants were devoid of symptoms; in contrast, non-primed plants exhibited small lesions with a length of 0.46 cm. The typical necrotic symptoms of the blast were observed at 48 hpi, which were further enhanced at 72 hpi (Figure 1A). After 6 days of inoculation, the lesion length was 2.10 cm in non-primed plants, whereas primed plants recorded 1.23 cm (Figure 1B).

### 3.2 Primary defense response through the production of ROS

**FIGURE 2:**
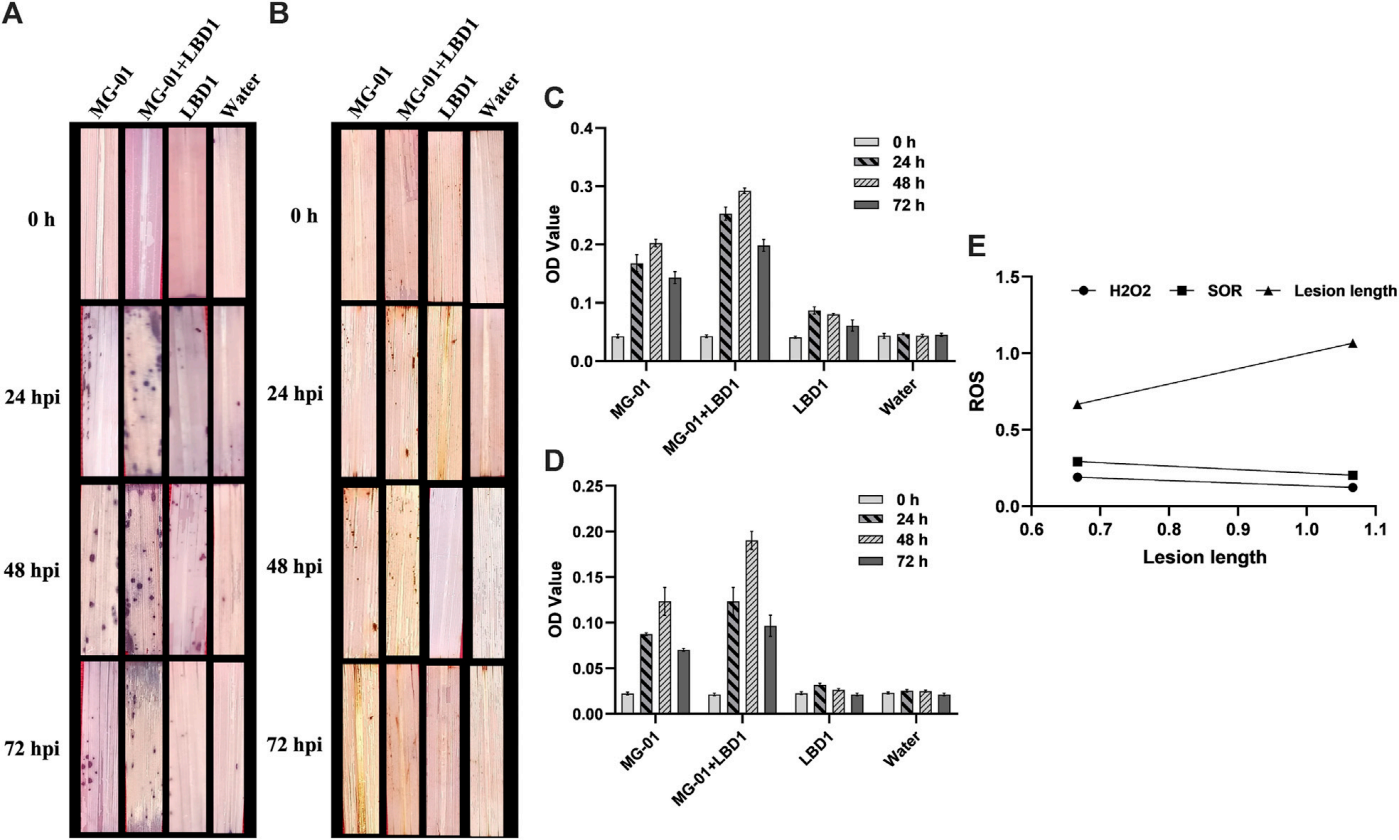
Induction of early defense through the production of ROS in primed plants at different time intervals. *In vivo* localization of superoxide radicals **(A)** and hydrogen peroxide **(B)** in response to MG-01 in primed and non-primed plants. Estimation of superoxide radicals **(C)** and hydrogen peroxide **(D)** at different intervals. Simple linear regression analysis **(E)** shows the correlation between superoxide radicals (SOR), hydrogen peroxide (H_2_O_2_), and lesion length.

We validated the role of ROS as a defense response by determining H_2_O_2_ and O_2_^−^ radicals. *In vivo* localization of these radicles indicated no noticeable differences with seaweed biostimulant and water-sprayed treatments (Figures 2A, B). However, when non-primed and challenge-inoculated plants were compared with biostimulant-primed plants, the ROS accumulation was significantly higher with respect to time intervals. At zero-hour, H_2_O_2_ and O_2_^−^ production ranged from 0.021 to 0.023 and 0.040-0.043 OD, respectively. Primed plants recorded fold changes of 7.90 and 5.83 at 48 hpi, whereas non-primed plants recorded 4.52 and 3.75-fold changes in the accumulation of H_2_O_2_ and O_2_^−^ respectively (2C and D). The relationship between the ROS and lesion length is negatively correlated, and the simple linear regression equations are mentioned in Figure 2E.

### 3.3 DEGs and gene ontology (GO) analysis

**FIGURE 3:**
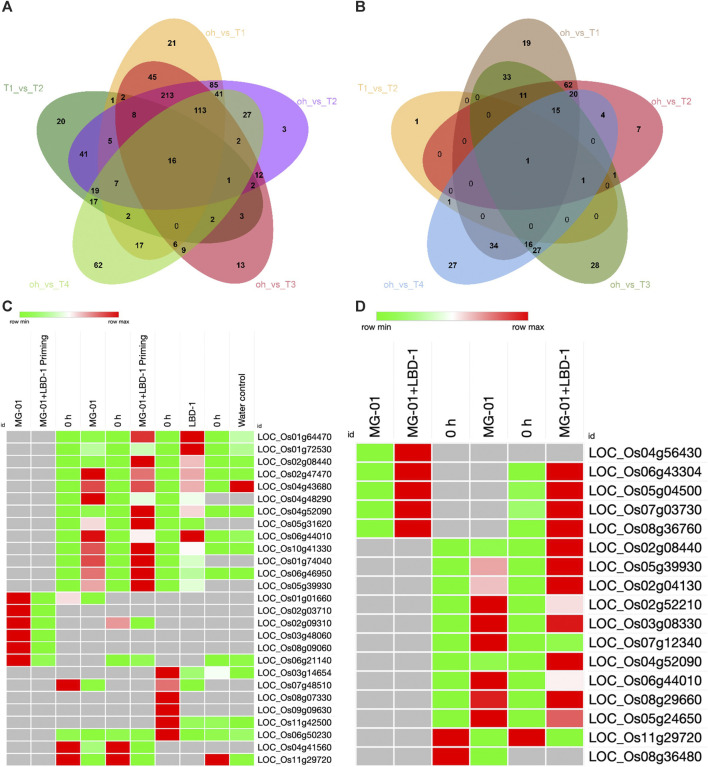
Differentially expressed genes in primed and non-primed rice plants. Venn diagram showing the common and unique genes upregulated **(A)** and downregulated **(B)** in the comparative analyses. Heat maps showing the commonly up and downregulated genes in different treatments **(C)** and Defense-related genes up and downregulated in primed and non-primed plants **(D)**.

The number of upregulated and downregulated genes varied across treatments at zero-hour and 48 hpi. A total of 3498 DEGs were identified, 1116 DEGs were explicitly regulated in pathogen-inoculated treatments, i.e., T_1_ and T_2_ (Supplementary Material S1). GO analysis was carried out to determine the functions of the DEGs. The analysis categorized the DEGs into biological processes, molecular processes, and cellular components (Supplementary Figure S1). A total of 787, 327, and 304 DEGs were involved in biological, cellular components, and molecular functions in comparative analysis between primed and non-primed rice plants (Figures 3A, B; Supplementary Figure S2). In LBD1-primed and challenged inoculated plants, a higher number of genes were regulated in cellular components. The number of up and downregulated genes varied across different treatments. Eighty-five genes were common to both zero *vs*. T_1_ and zero *vs*. T_2,_ while 19 and 7 DEGs were uniquely expressed in both comparisons. In pathogen-inoculated treatments, i.e., T_1_ and T_2_, 62 DEGs were commonly downregulated (Figures 3A, B). Heat maps depicting the up and down regulating genes in different treatments are illustrated in Figures 3C, D.

### 3.4 Plant Gene Sequence Enrichment Analysis (PlantGSEA)

To interpret the biological meaning of genes by computing the overlaps with previously defeined gene sets the PlantGSEA was performed. The comparitive results of PlantGSEA in each treatments are shown in Supplementary Material S2. There was a surge in number of genes for the synthesis secondary metabolite, plant hormone signal transduction, and phytoalexin production in primed and challenge inoculated plants (T_2_). A total of 699 and 144 genes were involved in biosynthesis of secondary metabolites and plant hormone signal transduction respectively. The genes responsible for Jasmonic acid (41), ethylene (18), salicylate (10), phenylpropanoid (11), oryzalexin (2), suberin (19) Diterpenoid (23) and flavonoid biosynthesis (17) were increased at 48 hpi which could be responsible for resistance against blast disease. The phytoalexins viz., oryzalexin A-F (LOC_Os12g30824) and oryzalexin S (LOC_Os11g28530) biosynthic genes were upregulated in T_2._ None of the defense-related genes were downregulated in primed plants (T_2_), whereas in non-primed (T_1_), a total of 31 genes responsible for phenylpropanoid biosynthesis were downregulated.

### 3.5 Comparative analysis of different treatments

#### 3.5.1 MG-01 inoculation *vs*. Priming + MG-01 infection (T_1_ *vs*. T_2_)

Comparison of differentially expressed genes between pathogen alone (T_1_) and Pathogen + LBD-1 (T_2_) resulted in the upregulation of 235 genes in T_2_. A total of 12 genes were downregulated; however, none were significant (*p* > 0.05). Most defense-related genes were upregulated in LBD-1 primed with challenged inoculation of MG-01. The cysteine-rich receptor-like protein kinase (LOC_Os04g56430), cytochrome P450 (LOC_Os06g43304), peroxidase precursor (LOC_Os05g04500), SCP-like extracellular protein (LOC_Os07g03730), remorin C-terminal domain containing protein (LOC_Os08g36760) were upregulated with fold change of 5.60, 4.22, 4.68. 5.58 and 4.70. These results are correlated with qPCR validation. Bet v I family protein (LOC_Os12g36880), cytochrome P450 (LOC_Os10g16974), chalcone synthase (LOC_Os11g32650) and O-methyltransferase (LOC_Os08g06100) were the important genes upregulated in T_2_ when compared withT_1_.

#### 3.5.2 Zero-hour *vs*. MG-01 inoculation (zero-hour *vs*. T_1_)

A total of 577 genes were upregulated in blast pathogen (MG-01) control (T_1_) compared to zero-hours. The FPKM values of all the differentially expressed genes were depicted in scattered plots (Supplementary Figure S2). The helix-loop-helix DNA-binding domain-containing protein (LOC_Os01g56690), phenylalanine ammonia-lyase (LOC_Os02g41670), peroxidase precursor (LOC_Os12g02060 and LOC_Os07g48020), harpin-induced protein 1 domain-containing protein (LOC_Os04g58850), NADP-dependent oxidoreductase (LOC_Os04g41960) and CHIT14—Chitinase family protein precursor (LOC_Os10g39680) were the important defense-related genes.

### 3.6 Regulation by transcription factors and signal transduction

Challenge inoculation of MG-01 in rice activated many AP2, MYB, and WRKY transcripts. In T_1,_ different types of AP2 domain containing proteins (LOC_Os09g28440, LOC_Os08g36920, LOC_Os07g42510), LOC_Os05g41780, LOC_Os10g41330, LOC_Os04g52090) were upregulated. MYB family transcription factors (LOC_Os05g35500, LOC_Os03g20090, and LOC_Os04g43680) were upregulated with log2 fold changes of 2.45, 3.91, and 5.69, respectively. The MYB transcription factor TaMYB1 (LOC_Os06g43090) is another transcript of the same family upregulated with a fold change of 3.76. The wound-induced protein WI12 (LOC_Os03g18770), WRKY1 (LOC_Os01g14440), WRKY53 (LOC_Os05g27730), WRKY74 (LOC_Os09g16510), WRKY7 (LOC_Os05g46020), WRKY24 (LOC_Os01g61080), WRKY28 (LOC_Os06g44010) and WRKY71 (LOC_Os02g08440) were also upregulated in T_1_. The protein kinases involed in the signal transduction *viz*., CAMK_CAMK_like.12—CAMK which includes calcium/calmodulin-dependent protein kinases (LOC_Os04g49510 and LOC_Os02g03410), CAMK_KIN1/SNF1/Nim1_like.4—CAMK including calcium/calmodulin-dependent protein kinases (LOC_Os11g02240) and CGMC_MAPKCGMC_2_ERK.2 - CGMC including CDA, mitogen-activated protein kinase (MAPK), GSK3, and CLKC kinases (LOC_Os03g17700) were upregulated in T_1_ which play an important role in MAPK or CAPK pathway during plant-pathogen interaction. A total of three calmodulin-binding proteins (LOC_Os12g36910, LOC_Os01g04280, LOC_Os12g36110, and LOC_Os01g38980), one calmodulin-like protein 1 (LOC_Os01g72080) and four Calmodulin-related calcium sensor proteins (LOC_Os01g72100, LOC_Os01g04330, LOC_Os05g31620, and LOC_Os01g72530) were upregulated in T_1_ with log2 fold change of 2.86, 3.42, 4.00, 3.50, 3.80, 3.91, 5.19 and 6.85 respectively.

#### 3.6.1 Zero-hour *vs*. LBD1 Priming + MG-01 inoculation (zero-hour *vs*. T_2_)

The phenylalanine ammonia-lyase (LOC_Os02g41670), NADP-dependent oxidoreductase (LOC_Os04g41960), terpene synthase (LOC_Os03g24690), chalcone synthase (LOC_Os11g32650), harpin-induced protein 1 domain-containing protein (LOC_Os04g58850) and beta-glucan-binding protein 4 (LOC_Os09g21210) were upregulated in T_2_. A total of three peroxidase precursors (LOC_Os07g48020, LOC_Os12g02060, LOC_Os04g59150) and two tyrosine-protein kinase domain-containing proteins (LOC_Os09g27010 and LOC_Os06g10160) were upregulated which play a role in up-stream of Salicylic acid (SA) signaling pathway.

### 3.7 Regulation by transcription factors signal transduction

A total of four AP2 domain-containing proteins (LOC_Os08g36920, LOC_Os09g28440, LOC_Os01g58420, and LOC_Os05g41780) and two AN1-like zinc finger domain-containing proteins (LOC_Os09g31200 and LOC_Os08g39450) were expressed in primed plants having a role in SA biosynthesis. The WRKY53 (LOC_Os05g27730), wound-induced protein WI12 (LOC_Os03g18770), WRKY74 (LOC_Os09g16510), WRKY24 (LOC_Os01g61080), and WRKY71 (LOC_Os02g08440) were upregulated in which WRKY71 play a vital role in the production of defense-related genes. Several other transcription factors, *viz*., The MYB family transcription factor (LOC_Os05g35500 and LOC_Os03g20090) MYB transcription factor TaMYB1 (LOC_Os06g43090) and two cytochrome P450 (LOC_Os09g26960 and LOC_Os03g55800) were upregulated. MYB TFs regulate the development, differentiation, and metabolism of plant cells and play a vital role in stress response. The STE_MEKK_ste11_MAP3K.18 - STE kinases includinghomologs to sterile 7, sterile 11, and sterile 20 from yeast (LOC_Os05g46750), CGMC_MAPKCGMC_2_ERK.2—CGMC including CDA, MAPK, GSK3 and CLKC kinases (LOC_Os03g17700) were upregulated. A total of four calmodulin-related calcium sensor proteins (LOC_Os05g13580, LOC_Os01g, 2100, LOC_Os01g04330, and LOC_Os05g31620), one calmodulin-like protein 1 (LOC_Os01g72080) and three calmodulin-binding protein (LOC_Os12g36110 LOC_Os01g38980 and LOC_Os01g04280) were upregulated in primed and challenge inoculated plants.

### 3.8 Defense-related enzymes, other TFs and growth regulating genes

A total of two chitinase genes were upregulated, i.e., CHIT3—Chitinase family protein precursor (LOC_Os04g41680) and CHIT14—Chitinase family protein precursor (LOC_Os10g39680), which plays an important role in defense by degrading the fungal cell wall. The GATA zinc finger domain-containing protein (LOC_Os02g56250), zinc finger, RING-type, putative (LOC_Os05g01940), SNARE domain-containing protein (LOC_Os02g24080), ZIM domain-containing protein (LOC_Os03g28940), ACT domain-containing protein (LOC_Os03g29980), PRAS-rich protein (LOC_Os01g28790), ZIM domain-containing protein (LOC_Os03g08330), EF-hand family protein (LOC_Os06g46950) and lrgB-like family protein (LOC_Os10g42780) were upregulated suggesting the role of these genes in defense mechanism. The protein kinase (LOC_Os01g06240), glycerol-3-phosphate acyltransferase 1 (LOC_Os01g22560), helix-loop-helix DNA-binding domain-containing protein (LOC_Os01g56690), RING-H2 finger protein ATL4O precursor (seed development) (LOC_Os02g35440), phosphate-induced protein 1 conserved region domain containing protein (LOC_Os02g51970), cysteine-rich repeat secretory protein 55 precursor (LOC_Os03g16960), terpene synthase, putative (LOC_Os03g24690) and peptide transporter PTR2 (LOC_Os04g50940) are the growth regulating genes which were upregulated in plants primed with seaweed bio-stimulant.

#### 3.8.1 Zero-hour *vs*. LBD1 (zero-hour *vs*. T_3_)

A total of 184 and 472 genes were down and upregulated, respectively. MYB family transcription factor (LOC_Os01g19330), WRKY26 (LOC_Os01g51690), WRKY67 (LOC_Os05g09020), WRKY1 (LOC_Os01g14440), and WRKY7 (LOC_Os05g46020) were upregulated, whereas WRKY112 (LOC_Os09g09630), peroxidase precursor (LOC_Os05g04500), dirigent (LOC_Os11g42500) and thioredoxin (LOC_Os07g48510) were downregulated. The transcripts involved in signal transduction viz., CAMK_CAMK_like.27- CAMK includes calcium/calmodulin-dependent protein (LOC_Os04g49510), CAMK_CAMK_like.12 - CAMK includes calcium/calmodulin-dependent protein kinases (LOC_Os02g03410), CAMK_KIN1/SNF1/Nim1_like.30—CAMK includes calcium/calmodulin-dependent protein kinases (LOC_Os07g48090) and CGMC_MAPKCGMC_2_ERK.2—CGMC includes CDA, MAPK, GSK3, and CLKC kinases (LOC_Os03g17700). A total of six calmodulin binding proteins (LOC_Os12g36910, LOC_Os12g36110, LOC_Os01g38980, LOC_Os12g36110, LOC_Os01g04280 and LOC_Os01g38980), two calmodulin-like proteins (LOC_Os01g72080 and LOC_Os01g72080) and eight calmodulin-related calcium sensor proteins (LOC_Os03g21380, LOC_Os05g13580, LOC_Os01g72100, LOC_Os05g13580, LOC_Os01g72100, LOC_Os05g31620, LOC_Os01g04330 and LOC_Os01g72530) were upregulated in T_3_ (Table 2).

**TABLE 2:** Up and downregulated genes in different treatments which are commonly or specifically expressed.

Gene ID	Gene name	Treatment/s	Up/downregulated
LOC_Os01g72530	OsCML31 - Calmodulin-related calcium sensor protein	T_1_, T_2_ and T_3_	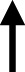
LOC_Os02g08440	WRKY71	T_1_, T_2_ and T_3_	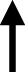
LOC_Os02g47470	Cytochrome P450, putative	T_1_, T_2_ and T_3_	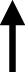
LOC_Os04g43680	MYB family transcription factor, putative	T_1_ and T_2_	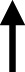
LOC_Os04g48290	MATE efflux family protein, putative, expressed	T_1_, T_2_ and T_3_	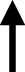
LOC_Os04g52090	AP2 domain containing protein	T_1_, T_2_ and T_3_	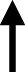
LOC_Os06g44010	WRKY28	T_1_ and T_3_	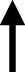
LOC_Os10g41330	AP2 domain containing protein	T_1_, T_2_ and T_3_	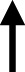
LOC_Os01g01660	Isoflavone reductase, putative	T_1_, T_2_ and T_3_	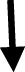
LOC_Os02g09310	Cytochrome P450, putative	T_1_, T_2_ and T_3_	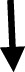
LOC_Os03g15370	Ubiquitin fusion protein, putative	T_1_, T_2_ and T_3_	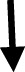
LOC_Os03g48060	Class I glutamine amidotransferase, putative	T_1_, T_2_ and T_3_	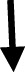
LOC_Os08g09060	Cupin domain containing protein	T_1_, T_2_ and T_3_	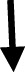
LOC_Os04g56430	Cysteine-rich receptor-like protein kinase	T_2_	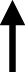
LOC_Os05g04500	Peroxidase precursor	T_2_	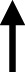
LOC_Os06g43304	Cytochrome P450	T_2_	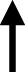
LOC_Os07g03730	SCP-like extracellular protein	T_2_	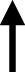
LOC_Os08g36760	Remorin C-terminal domain containing protein	T_2_	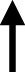
LOC_Os02g08440	WRKY71	T_1_ and T_2_	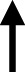
LOC_Os05g39930	Spotted leaf 11	T_1_ and T_2_	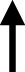
LOC_Os02g04130	DUF1645 domain containing protein, putative	T_1_ and T_2_	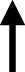
LOC_Os02g52210	Zinc finger, C3HC4 type domain containing protein	T_1_ and T_2_	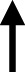
LOC_Os03g08330	ZIM domain containing protein, putative	T_1_ and T_2_	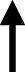
LOC_Os07g12340	NAC domain-containing protein 67, putative	T_1_ and T_2_	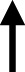
LOC_Os04g52090	AP2 domain containing protein	T_1_ and T_2_	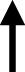
LOC_Os06g44010	WRKY28	T_1_ and T_2_	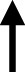
LOC_Os08g29660	WRKY69	T_1_ and T_2_	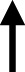
LOC_Os05g24650	DUF567 domain containing protein, putative	T_1_ and T_2_	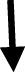
LOC_Os11g29720	Cytochrome P450, putative	T_1_ and T_2_	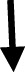
LOC_Os08g36480	Nitrate reductase, putative	T_1_ and T_2_	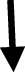

T_1_, MG-01; T_2_, MG-01 + LBD1; T_3_, LBD1; 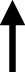, Upregulated and 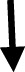, Downregulated.

#### 3.8.2 Other defense and growth-promoting related genes

Class I glutamine amidotransferase (LOC_Os04g38950) and zinc finger family protein (LOC_Os05g03760) are the structural and regulatory proteins that were significantly upregulated. A total of three harpin-induced protein 1 domain-containing proteins (LOC_Os12g06220, LOC_Os04g58850, and LOC_Os01g64470), one ZIM domain-containing protein (LOC_Os03g28940), SNARE domain-containing protein (LOC_Os02g24080), GATA zinc finger domain-containing protein (LOC_Os02g56250), disease resistance RPP13-like protein 1 (LOC_Os03g20840), NADP-dependent oxidoreductase (LOC_Os04g41960), SRC2 protein (LOC_Os01g70790) and NB-ARC/LRR disease resistance protein (LOC_Os04g43440) each were upregulated. Growth-promoting genes *viz*., inorganic phosphate transporter (LOC_Os03g05620), gibberellin response modulator protein (LOC_Os07g39470), tyrosine-protein kinase domain-containing protein (LOC_Os06g10160), DnaK family protein (LOC_Os03g16860), BURP domain-containing protein (LOC_Os10g26940) and AMP-binding domain-containing protein (LOC_Os04g58710) were upregulated in plants treated with seaweed biostimulants (T_3_).

#### 3.8.3 Validation of transcripts using qRT-PCR

**FIGURE 4:**
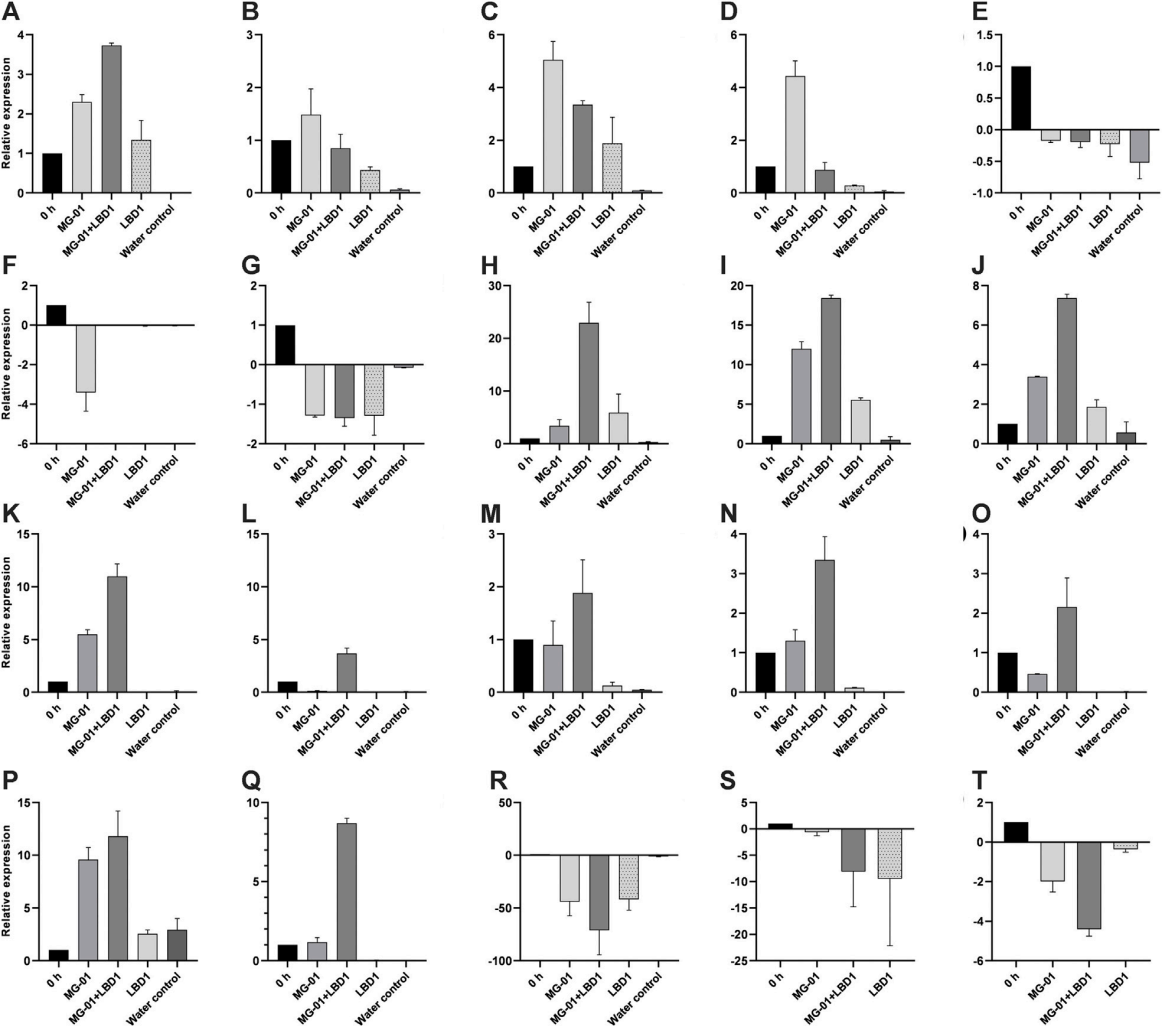
qPCR validation of selected differentially expressed genes in different treatments. LOC_Os01g72530 **(A)**, LOC_Os02g08440 **(B)**, LOC_Os02g47470 **(C)**, LOC_Os04g48290 **(D)**, LOC_Os01g01660 **(E)**, LOC_Os03g15370 **(F)**, LOC_Os08g09060 **(G)**, LOC_Os04g56430 **(H)**, LOC_Os06g43304 **(I)**, LOC_Os05g04500 **(J)**, LOC_Os08g36760 **(K)**, LOC_Os02g08440 **(L)**, LOC_Os02g04130 **(M)**, LOC_Os03g08330 **(N)**, LOC_Os07g12340 **(O)**, LOC_Os06g44010 **(P)**, LOC_Os04g52090 **(Q)**, LOC_Os08g36480 **(R)**, LOC_Os05g24650 **(S)**, and LOC_Os11g29720 **(T)**.

We selected 20 genes obtained from RNA-Seq, including up and downregulated genes based on their role in defense mechanisms and biological functions for validation. Expression study of all the genes showed a strong correlation with RNA-Seq data. Relative expressions of the validated genes at different time points are depicted in Figure 4.

#### 3.8.4 Effect of seaweed biostimulants in the management of blast disease and yield in micro plot experiment

**FIGURE 5:**
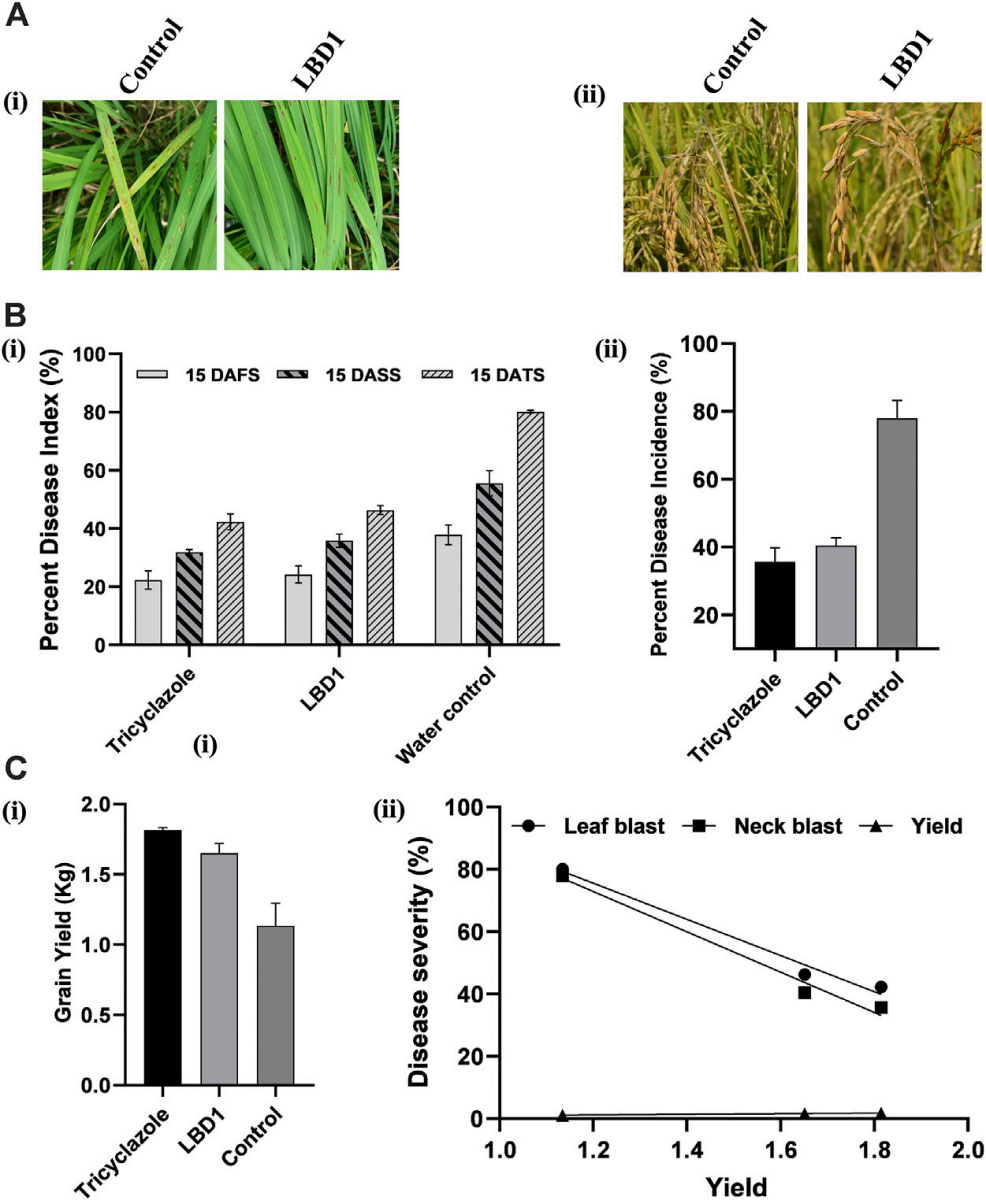
Effect of seaweed biostimulant on leaf and neck blast disease in micro plot experiment. Representative images **(A)** of the leaf blast **(Ai)** and neck blast **(Aii)** in control and LBD1 sprayed plots. Percent disease index (PDI) **(B)** of leaf blast **(Bi)** and PDI of neck blast **(ii)** in different treatments. Grain yield in different treatments **(Ci)** and simple linear regression showing the correlation between yield, leaf, and neck blast **(Cii)**.

In a micro plot experiment, the effect of seaweed bioformulation on rice was evaluated by measuring different parameters, viz., Leaf blast, neck blast, and grain yield. Water inoculated plots recorded the highest PDI of 80.16% and 77.97% leaf and neck blast after 15 Days of third spray (DATS) and at harvest, respectively. In biostimulant sprayed plots, up to 46.13% and 40.46% reduction over control was observed with respect to leaf and neck blast disease PDI (Figures 5A, B). The highest straw and grain yield was recorded in tricyclazole-treated plots with 1.80 Kg and 1.81 Kg, respectively; however, there was no significant difference with the seaweed-treated plots (Figure 5C). The negative coefficient in simple linear regression suggested that increased leaf and neck blast PDI reduces the grain yield (Figure 5D).

## 4 Discussion

In agriculture, applying seaweed-derived biostimulants is one of the recent strategies to boost crop yields through the enhancement of various metabolic processes, promoting tolerance/resistance to various abiotic and biotic stresses (Ali et al., 2022). Previous reports have confirmed that seaweed biostimulants act indirectly on plant’s metabolism by triggering several signaling cascades that initiate responses leading to the alleviation of various stresses and, consequently, enhanced growth and performance (Saeger et al., 2020; Banakar et al., 2022). Considering the devastating nature of rice blast disease, environmentally sustainable methods are more appealing in light of the public concern about using chemical fungicides in agricultural production (Sahana et al., 2022) *Kappaphycus alvarezii* seaweed extracts have been known for their plant biostimulant and stress alleviation activities on various crops (Kumar et al., 2019). However, very few reports are available showing its impact at the molecular level, which is crucial in identifying the mechanism of action of seaweed biostimulants on plants (Trivedi et al., 2021). RNA sequencing provides precise knowledge of the transcriptional changes induced by seaweed biostimulants in plants upon challenge infection with a pathogen.

The current study focused on profiling the rice transcriptome, which provided an in-depth view of molecular signals during rice-*M. oryzae* interaction. In glasshouse conditions, the infection of MG-01 led to the initiation of the blast disease symptoms at 24 hpi in non-primed plants. The size of the lesions enlarged with a time course and reached the maximum at 6 dpi. On the contrary, priming of the seaweed biostimulant restricted the further spread of the fungi leading to confined lesions. This primary defense in primed plants is attributed to the accumulation of ROS. Production of both superoxide radicles and hydrogen peroxide played a pivotal role in defense against blast disease in rice (Kumar et al., 2021).

A total of 3498 differentially expressed genes (DEGs) were identified; 1116 DEGs were specifically regulated in pathogen-inoculated treatments (T_1_ and T_2_). Functional analysis showed that most DEGs were involved in metabolism, transport, signalling, and defense. Expression levels of defense and growth-related genes were compared with primed and non-primed plants, which were challenge inoculated with MG-01. Widely distributed compounds in plant defense system are flavonoids and phenylpropanoids which possess different mode of action. The previous research have demonstrated the role of secondary metabolites in plant defense against pathogens (Zaynaba et al., 2018). A total of 86 and 14 genes were involved in biosynthesis phenylpropanoids and flavonoids respectively in LBD1 primed plants. Oryzalexins are the diterpenoid phytoalexins which participate in the defense against the pathogens in rice (Wang et al., 2018). The current study demonstrate the upregulation of LOC_Os12g30824 and LOC_Os11g28530 which play a crucial role in biosynthesis of oryzalexin A-F and oryzalexin S respectively. Suberin protects the host plant against the pathogen infection by reinforcing a mechanical barrier for the invasion and growth (Singh et al., 2021). A total of 19 genes were upregulated in T_2_ which have role in suberin biosynthesis.

**FIGURE 6:**
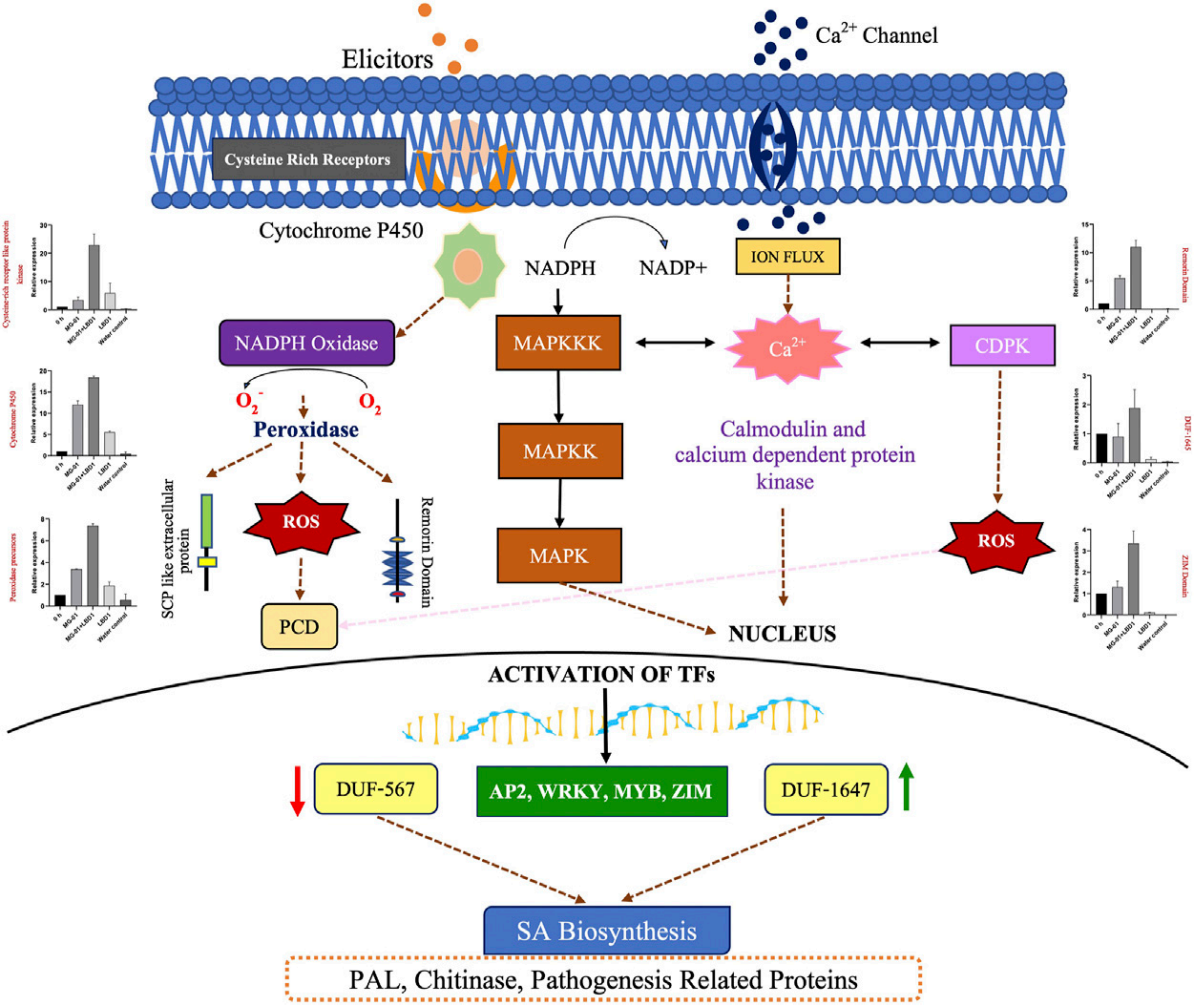
Overview of the mechanism involved in defense against blast disease. A model represents the pathways activated to induce resistance against MG-01 in seaweed-primed rice plants. The recognition of elicitors and the receptors trigger the generation of NADPH molecules, subsequently leads to the production ROS after oxidation. This stimulus activates the kinase signaling molecules involed in signal transduction. These molecules activates the transcription factors (WRKY, MYB, and ZIP) which altogether results in the activation of the defence related genes.

In a nutshell, priming of rice plants with seaweed bioformulations increased the expression of DEGs involved in various processes against MG-01. To explain the role of LBD1 in imparting the defense against blast disease in rice, we proposed a model depicting the crucial pathways involved (Figure 6). Signaling pathways play a crucial role in imparting resistance against pathogens in plants. This aids in activating and maintaining the innate defense in plants involving receptor-like kinases (MAPKs) and leucine-rich repeats (LRR) signalling (Ma et al., 2022). Several transcription factors like AP2, MYB, and WRKY activate the defense-responsive genes after perceiving the signals from defense signaling pathways in plants (Gene et al., 2017; Sureshkumar et al., 2019). Agarwal et al., 2016 reported a higher transcript level of pathogenesis-related genes due to *Kappaphycus* seaweed extract (KSWE) application under biotic stress in tomato seedlings. MPK2 (mitogen-activated kinase pathway gene) and Pti4 (transcription factor) were elevated in *Macrophomina*-affected tomato plants treated with KSWE, resulting in increased stress tolerance compared to its control. In another study, Patel et al., 2020 reported increased expression of antioxidant genes, MAP kinase genes, and stress-responsive WRKY transcription factors in KSWE-treated wheat plants. MAPK, as a signaling molecule, helps in the transduction of extracellular stimulation of cells into intra-cellular response leading to the activation of TFs in plants against pathogen invasion (Kumar et al., 2021).

The beta-D-xylosidase (LOC_Os04g54810), which is a putative gene that helps in secondary cell wall reinforcement, was downregulated in T_1_ [blast pathogen (MG-01) control]. In contrast, it was upregulated in T_2_ (Pathogen + LBD-1), which has a significant role in defense. Phenylalanine ammonia-lyase (LOC_Os02g41650), pathogenesis-related Bet v I family protein (LOC_Os12g36880), and chalcone synthase (LOC_Os11g32650) were upregulated in LBD-1 primed and challenged inoculated leaves when compared to non-primed plants. Phenylalanine ammonia-lyase (PAL) is a key enzyme in the phenylpropanoid pathway. PAL is also involved in synthesizing phytoalexins and lignin to prevent cell wall penetration by the pathogen (Bagal et al., 2012), and pathogenesis-related Bet v I family protein acts upstream of SA signaling mechanism. Chalcone synthase plays an important role in flavonoid biosynthesis (Dao et al., 2011), indicating the role of LBD-1 in imparting the defense against MG-01. Cytochrome P450 helps in the growth and development of plant cells, which was downregulated in pathogen-treated plants; however, it was upregulated in T_2_ (Pathogen + LBD-1) plants, indicating the role of LBD-1 in repairing the damaged cell due to pathogen attack. LTPL124 - Protease inhibitor/seed storage/LTP family protein precursor (LOC_Os03g01300) is a lipid transfer protein with eight conserved cysteine residues and represents the plant defensins. These are the cysteine-rich anti-microbial peptides involved in plant defense mechanisms. This gene is involved in disrupting the pathogen’s membrane via specific and non-specific electrostatic and hydrophobic interactions with cell surface group (Shai, 2002; Thevissen et al., 2003), ribosomal inactivation and protein inhibition (Ge et al., 2003). It was downregulated in T_1_ (blast pathogen (MG-01) control) and upregulated in T_2_ (Pathogen + LBD-1), which shows the role of LBD-1 in imparting the defense by disruption of the fungal membrane during the establishment of the pathogen.

Overexpression of OsCML16 calmodulin-related calcium sensor protein enhances resistance to rice blast disease, and the constitutive accumulation of OsCML 16 protein prepares rice plants for a rapid and potentiated defense response (Kishi-kaboshi et al., 2010). Calcium-dependent protein kinases (CDPKs/CPKs) and calcium-related proteins play an important role in plant defense. Since plants are exposed to a variety of stress, increasing intracellular Ca^2+^ levels results in a change in the level of H_2_O_2_ through binding to CaM/CML, a ubiquitous calcium-binding protein (Zhang et al., 2016). CaM/CML was discovered to be important for defense response in the plant by interacting with MLO (Mildew resistant locus O) (Kim et al., 2002). This suggests the potential communication between plant abiotic responses and immunity via signaling from MLO to Ca^2+^-dependent CaM/CML, which stimulates the production of H_2_O_2_ and leads to a defense mechanism.

Mitogen-activated protein kinase cascades, *i.e.,* MAP3K.18 (LOC_Os05g46750) represent candidates for downstream signaling processes during fungal infection. A MAPK cascade involving OsMKK4 and the MAPKs OsMPK3 and OsMPK6 was shown to transducer chitin elicitor signal in rice defense responses (Kishi-kaboshi et al., 2010). Transcription factors (TFs) play important roles in regulating defense-responsive genes. The WRKY, AP2/ERF, and MYB families have been reported to be involved in regulating rice defense responses to *M. oryzae* (Licausi et al., 2013; Yokotani et al., 2014). AP2 domain contains many amino acids and is part of the Ethylene Responsive Element (ERE), which helps in the induction of defense-related genes (Rashid et al., 2012). WRKY, AP2, and MYB family TFs were regulated in pathogen-inoculated treatments. WRKY 28 was specifically upregulated in T_1_ (Pathogen control), which was not regulated in T_2_. WRKY 24 and 71 were upregulated in both treatments. Previous studies have shown that WRKY 24 and 71 positively regulate rice defense response to *M. oryzae,* whereas WRKY28 showed negative regulation (Cheng et al., 2015).

Besides understanding the biochemical and molecular mechanism in blast-rice interaction, the present study also focused on the micro plot experiment in natural conditions revealing the role of seaweed biostimulants in managing the disease and increasing the grain yield. This suggested that LBD-1 induces resistance against MG-01 in the primed plants before challenge inoculation. Oligosaccharides and carrageenans present in the seaweed bioformulations are known to induce plant defense responses by modulating the activity of different defense pathways, including salicylate, jasmonate, and ethylene signalling pathways (Ali et al., 2016).

## 5 Conclusion

Seaweed extracts are known for their bio-stimulatory and stress alleviation activities in crop plants. In this study, transcriptome analysis unravels the gene expression patterns that impart the defense in seaweed-primed plants against blast disease. The induced defense is characterized by the production of ROS, upregulation of the genes involved in transcription regulation, signal transduction, secondary metabolite synthesis, and cell wall modification. WRKY, AP2/ERF, MYB, chitinases, and peroxidase were expressed predominantly in seaweed-primed plants. Thus, our study showed several genes and pathways activated in rice-*Magnaporthe* interaction, specifically in seaweed-primed plants leading to the defense.

## Data Availability

The data presented in the study are deposited in the NCBI repository with the accession numbers: SRR21600810; SRR21600809; SRR21600808; SRR21600807; SRR21600806; SRR21600805.

## Supplementary material

SUPPLEMENTARY FIGURE S1 Gene ontology for the differentially expressed genes in comparative analysis of different treatments. T_1_ *vs*. T_2_ **(A)** zero hours *vs*. T_1_ **(B)** zero hours *vs*. T_2_ **(C)** zero hours *vs*. T_3_ **(D)** and zero hours *vs*. T_4_ **(E)**.

SUPPLEMENTARY FIGURE S2 Scatter Plots representing up and down-regulated differentially expressed genes in different comparative analyses. T_1_ *vs*. T_2_ **(A)** zero hours *vs*. T_1_ **(B)** zero hours *vs*. T_2_ **(C)** zero hours *vs*. T_3_ **(D)** and zero hours *vs*. T_4_ **(E)**.

SUPPLEMENTARY MATERIAL S1
**(A)** Up-regulated genes in the comparitive analysis of all the treatment combinations. **(B)** Down-regulated genes in the comparitive analysis of all the treatment combinations **(C)** Total number DEG’s expressed, number reads and aligned percentage in each treatments.

SUPPLEMENTARY MATERIAL S2
**(A)** PlantGSEA for MG-01 *vs*. MG-01 + LBD1 **(B)** PlantGSEA for zero h vs MG-01 **(C)** PlantGSEA for zero h *vs*. MG-01 + LBD1 **(D)** PlantGSEA for zero h *vs*. LBD1 **(E)** PlantGSEA for zero h *vs*. Water control.
