# Supplementary figures and images for: Rice-Magnaporthe transcriptomics reveals host defense activation induced by red seaweed-biostimulant in rice plants
Source: Front Genet. 2023 Jun 23;14:1132561. doi: 10.3389/fgene.2023.1132561 (PMC10327602; doi:10.3389/fgene.2023.1132561)

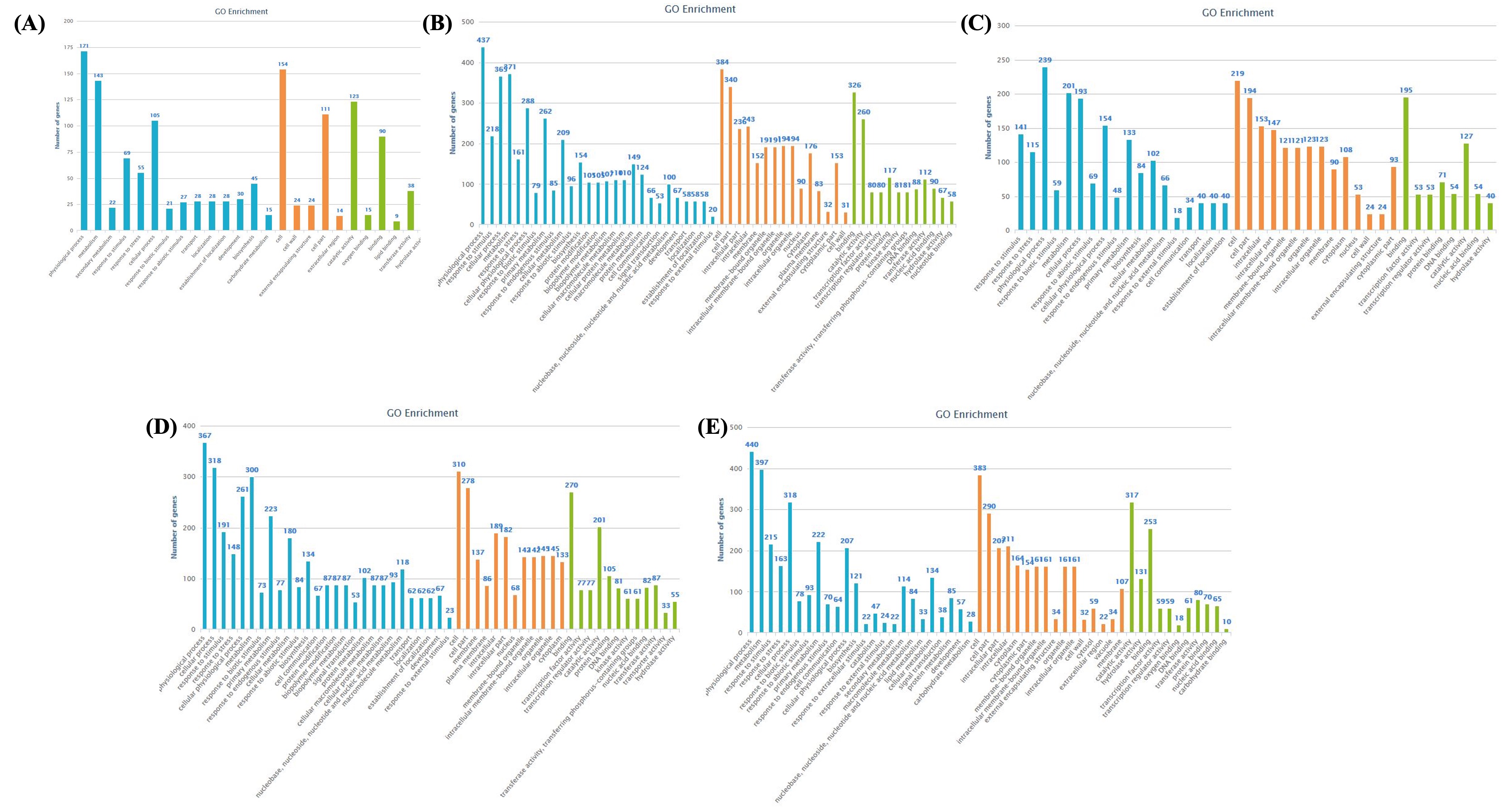

Supplement: Supplementary file 1 [file Image1.JPEG]

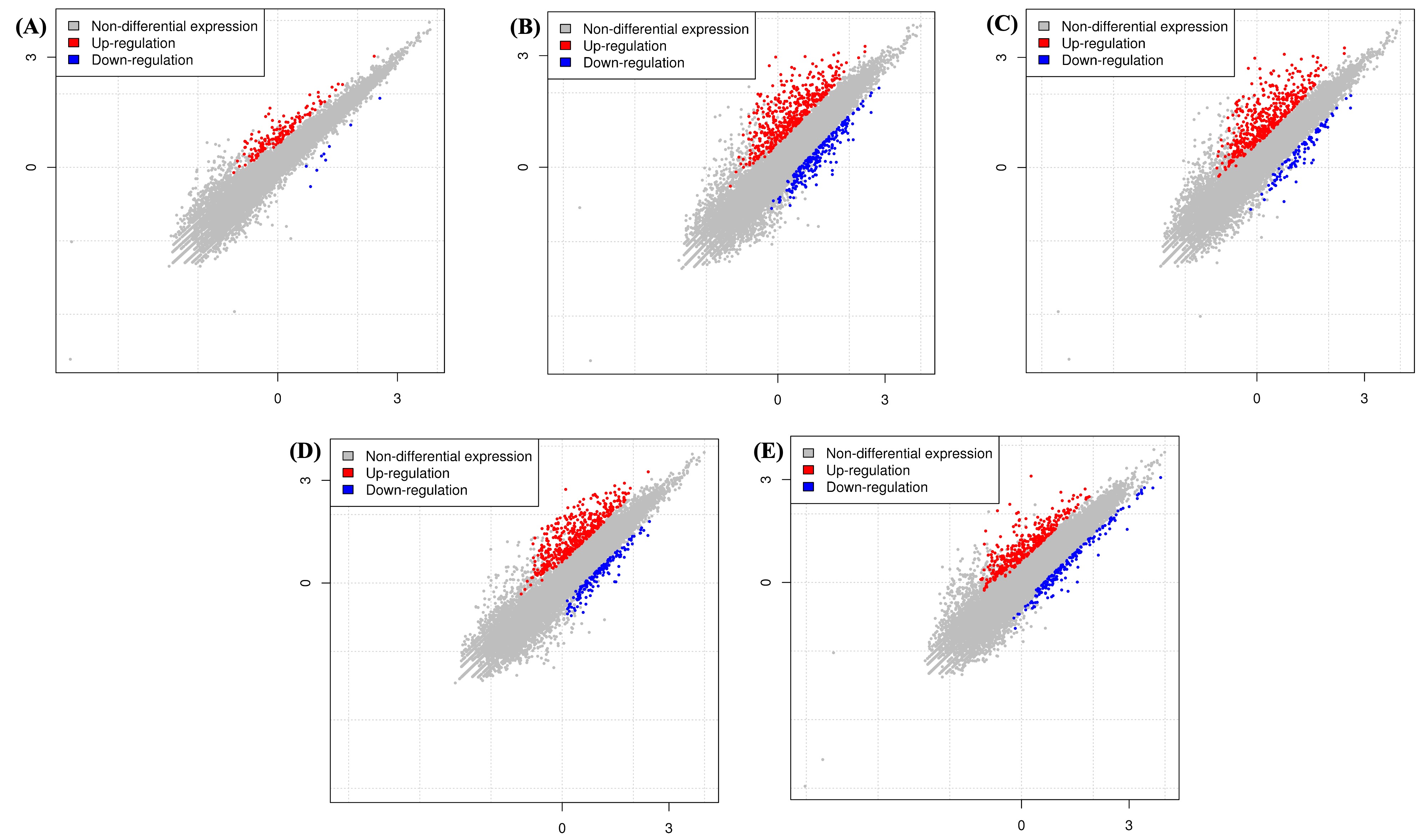

Supplement: Supplementary file 3 [file Image2.JPEG]
